# RETRACTION: Bioanalytical Evaluation of Wound Depth and Musculoskeletal Injuries: Synergizing Focused Assessment With Sonography for Trauma With Computed Tomography/Magnetic Resonance Imaging in Orthopaedic Trauma Care

**DOI:** 10.1111/iwj.70521

**Published:** 2025-04-18

## Abstract

**RETRACTION:** XueZ. and WenB. , “Bioanalytical Evaluation of Wound Depth and Musculoskeletal Injuries: Synergizing Focused Assessment With Sonography for Trauma With Computed Tomography/Magnetic Resonance Imaging in Orthopaedic Trauma Care,” International Wound Journal 21, no. 1 (2024): e14647, 10.1111/iwj.14647.38272795 PMC10805531

The above article, published online on 23 January 2024, in Wiley Online Library (http://onlinelibrary.wiley.com/), has been retracted by agreement between the journal Editor in Chief, Professor Keith Harding; and John Wiley & Sons Ltd. Following an investigation by the publisher, all parties have concluded that this article was accepted solely on the basis of a compromised peer review process. The editors have therefore decided to retract the article. The authors did not respond to our notice regarding the retraction.

**RETRACTION:** Z. Xue and B. Wen , “Bioanalytical Evaluation of Wound Depth and Musculoskeletal Injuries: Synergizing Focused Assessment With Sonography for Trauma With Computed Tomography/Magnetic Resonance Imaging in Orthopaedic Trauma Care,” International Wound Journal 21, no. 1 (2024): e14647, 10.1111/iwj.14647.38272795 PMC10805531

The above article, published online on 23 January 2024, in Wiley Online Library (http://onlinelibrary.wiley.com/), has been retracted by agreement between the journal Editor in Chief, Professor Keith Harding; and John Wiley & Sons Ltd. Following an investigation by the publisher, all parties have concluded that this article was accepted solely on the basis of a compromised peer review process. The editors have therefore decided to retract the article. The authors did not respond to our notice regarding the retraction.

